# Supplementary material for: Structure of the human outer kinetochore KMN network complex
Source: Nat Struct Mol Biol. 2024 Mar 8;31(6):874–83. doi: 10.1038/s41594-024-01249-y (PMC11189301; doi:10.1038/s41594-024-01249-y)

Source Data of Full-length, Uncropped Gels

Extended Data Figure 1

a

MIS12C

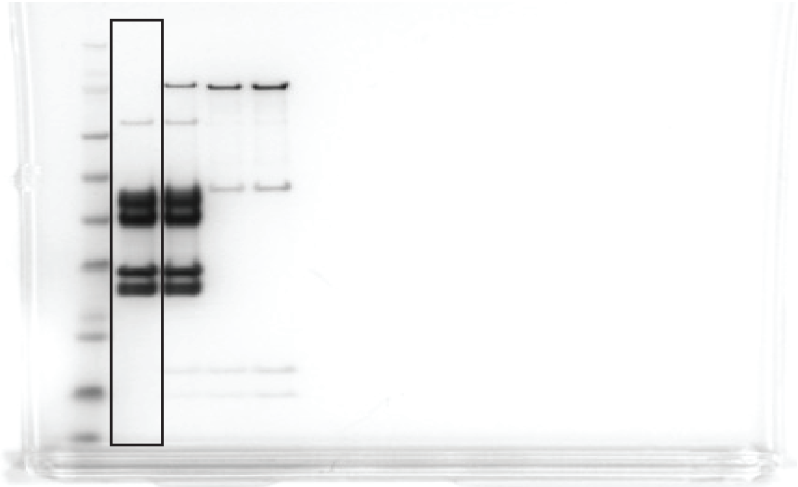

NDC80C

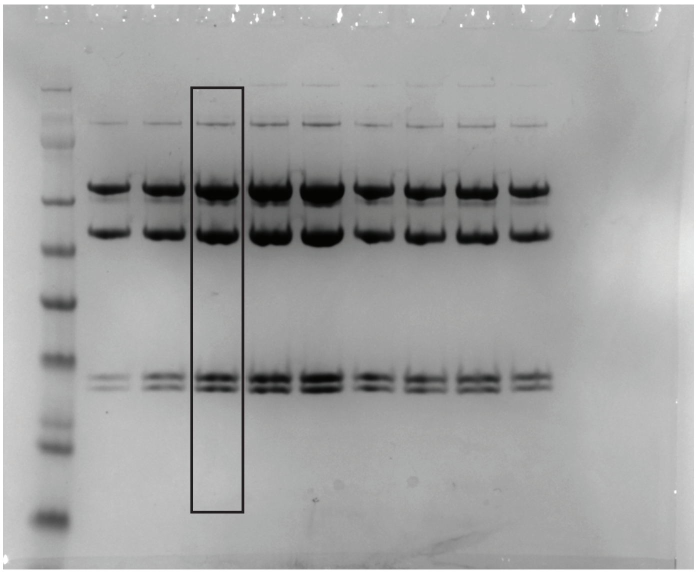

KNL1C

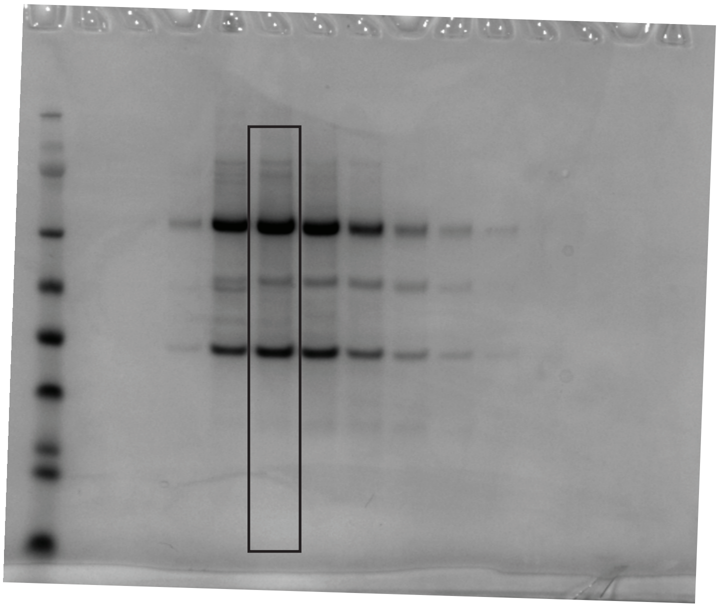

c

KMN

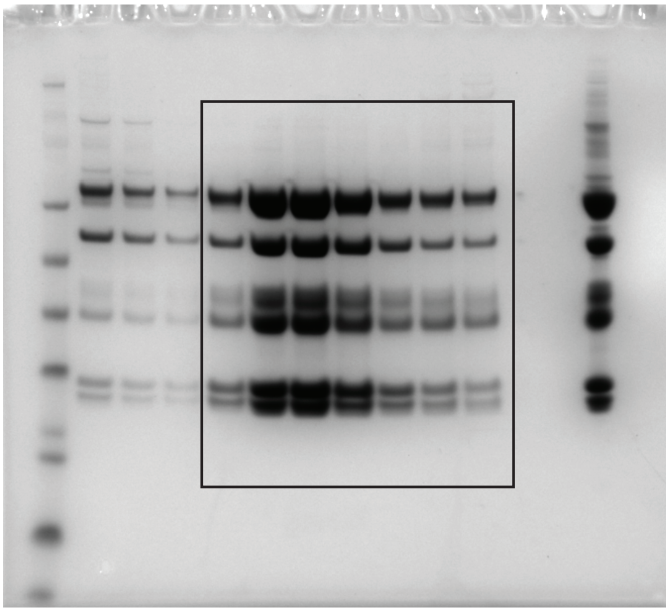

Source Data of Full-length, Uncropped Gels

Extended Data Figure 5

a

MIS12C
CENP-C$^{1\text{-}71}$-MBP

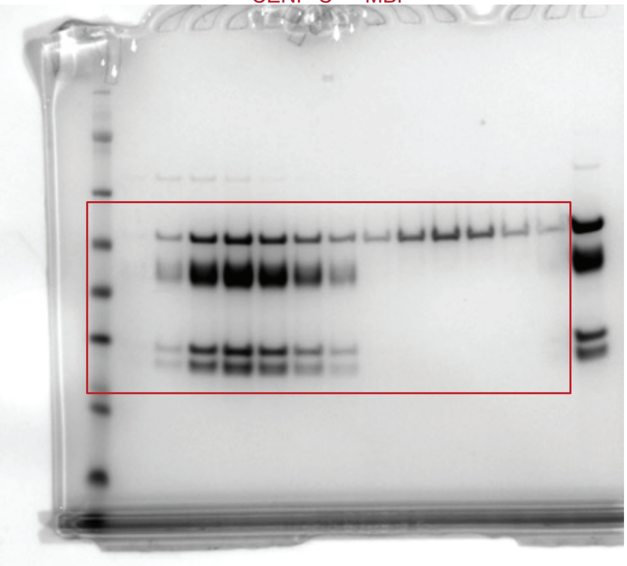

MIS12C$^{Dsn1\Delta N}$
CENP-C$^{1\text{-}71}$-MBP

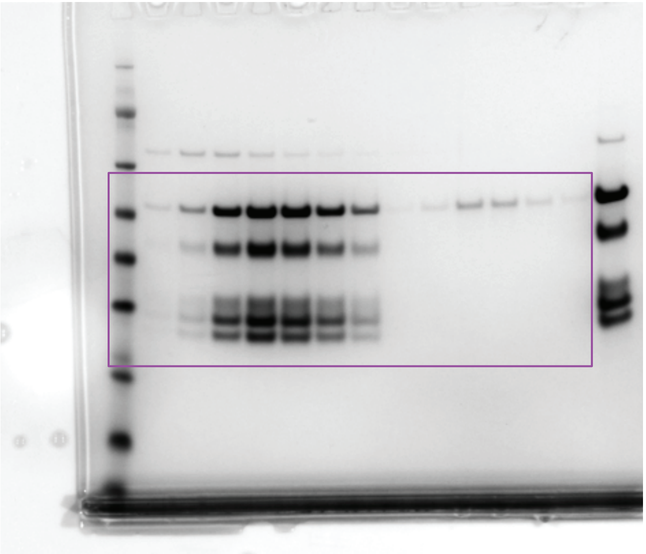

MIS12C$^{Dsn1\ W96A/R97A/R98A}$
CENP-C$^{1\text{-}71}$-MBP

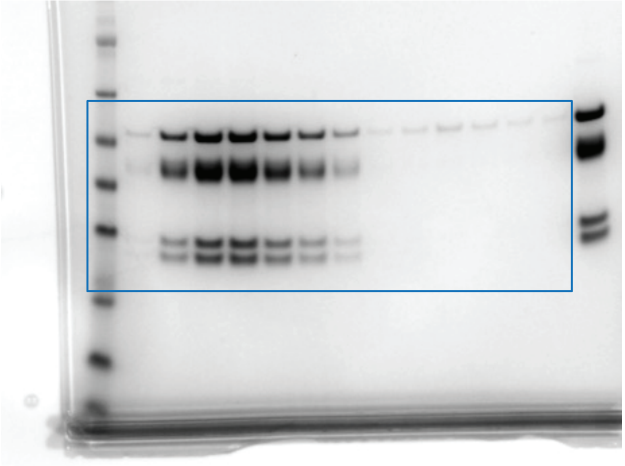

MIS12C$^{Dsn1\ S100D/S109D}$
CENP-C$^{1\text{-}71}$-MBP

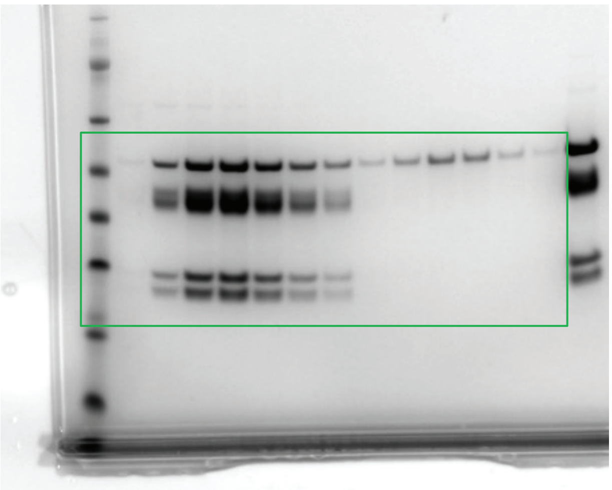

MIS12C

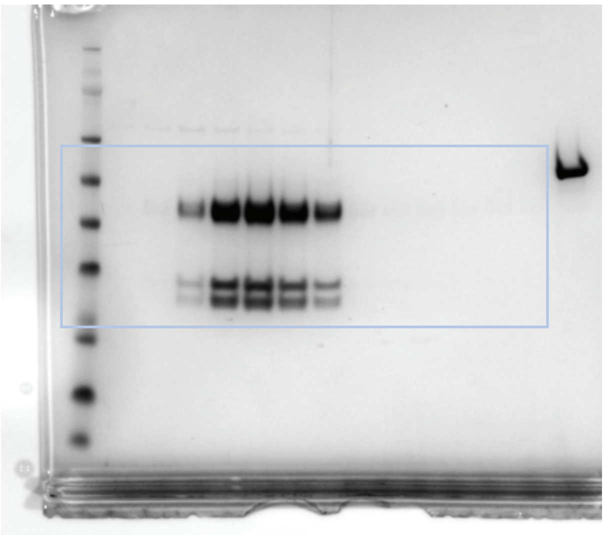

CENP-C$^{1\text{-}71}$-MBP

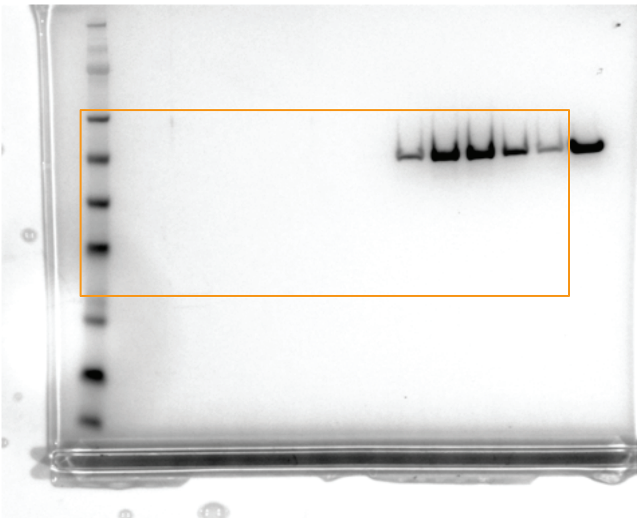

Source Data of Full-length, Uncropped Gels

Extended Data Figure 7

a

NDC80C

MIS12C

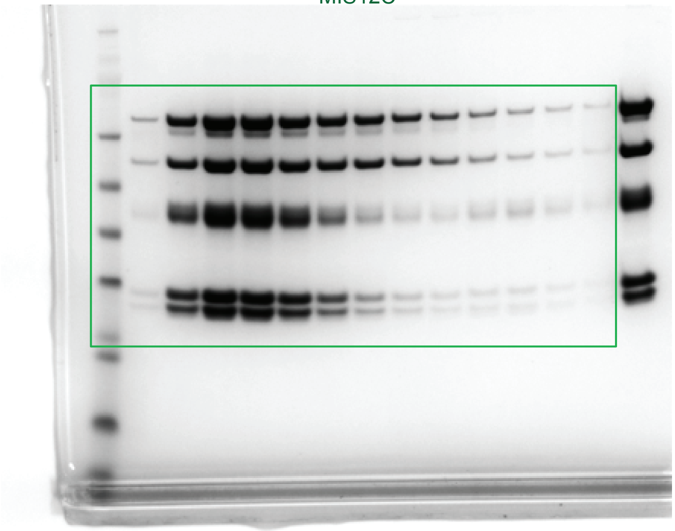

NDC80C

MIS12C$^{Nsl1\ E219R/V220R/F221A}$

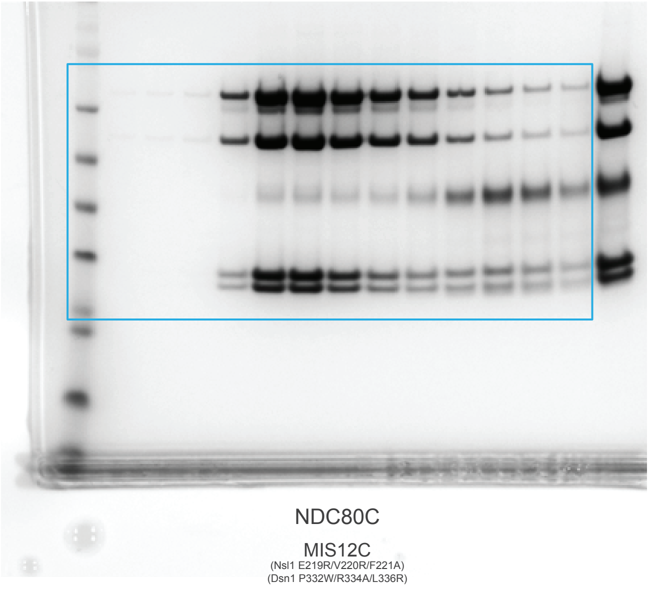

NDC80C

MIS12C$^{Dsn1\ P332W/R334A/L336R}$

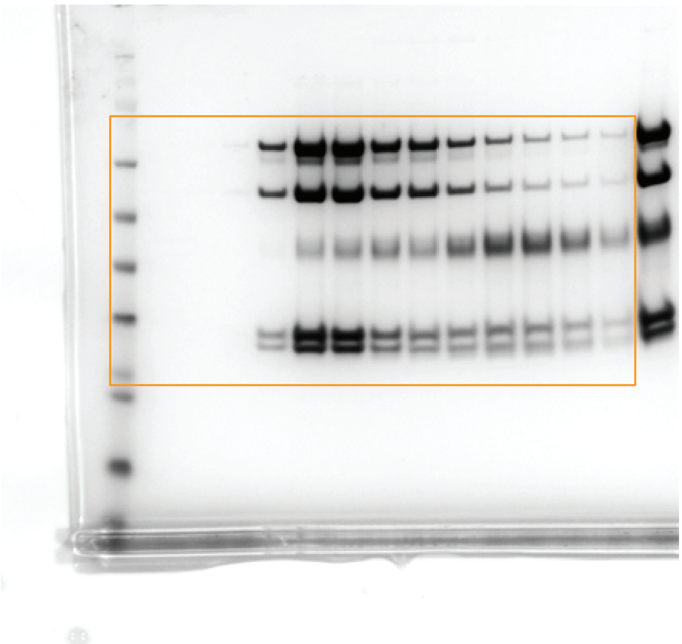

NDC80C

MIS12C

(Nsl1 E219R/V220R/F221A)

(Dsn1 P332W/R334A/L336R)

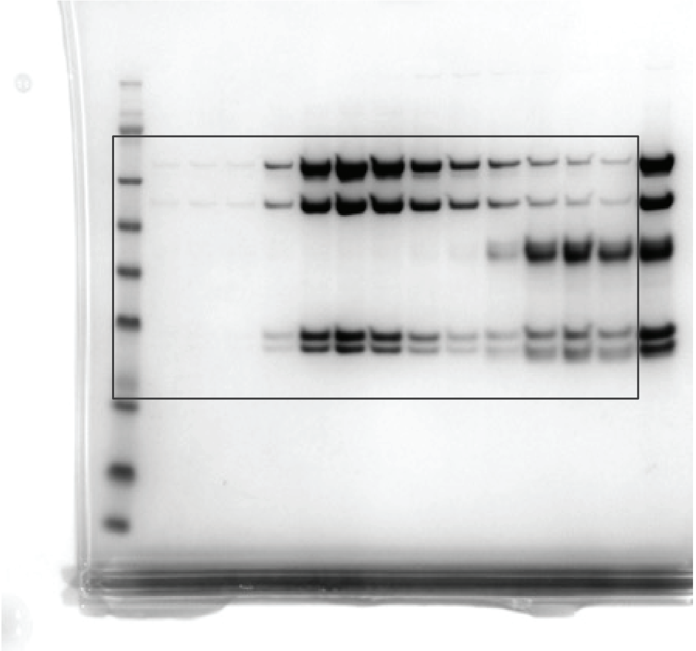

NDC80C

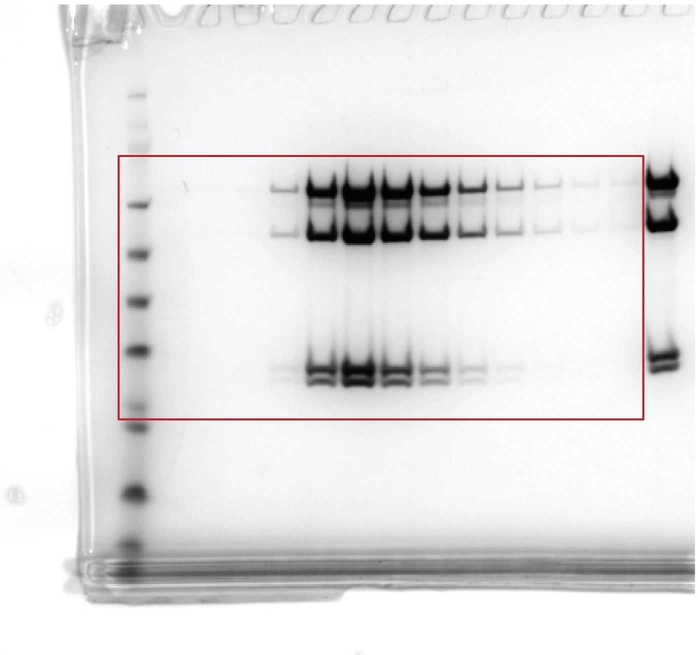

MIS12C

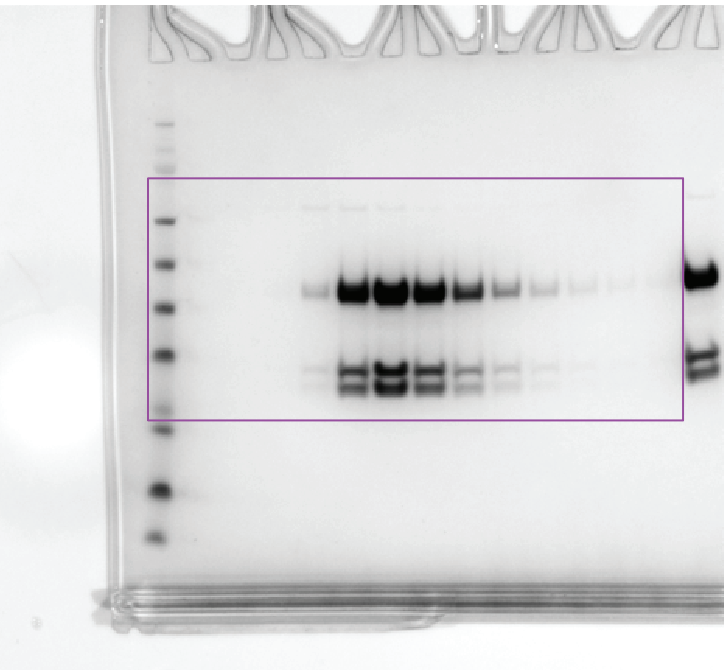

Source Data of Full-length, Uncropped Gels

Extended Data Figure 9

a

MIS12C
Knl1

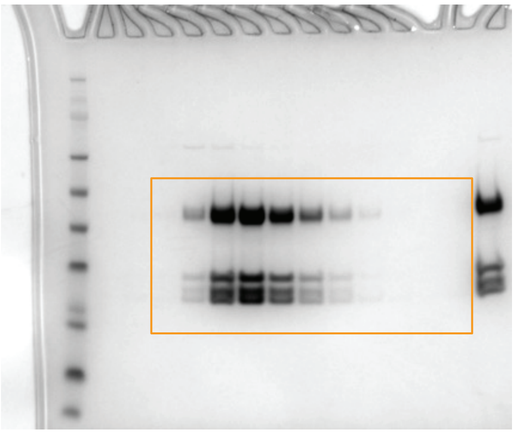

MIS12C
$Knl1^{mut1}$

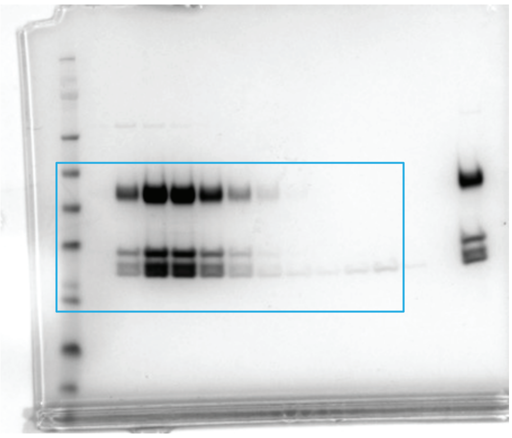

MIS12C
$Knl1^{mut2}$

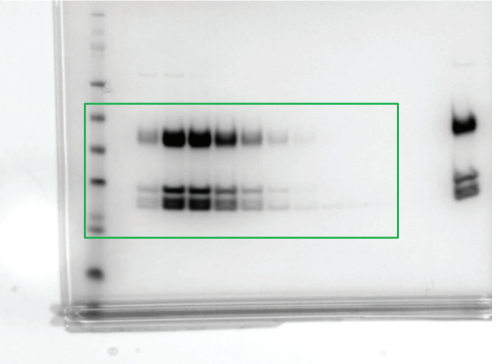

MIS12C
$Knl1^{mut3}$

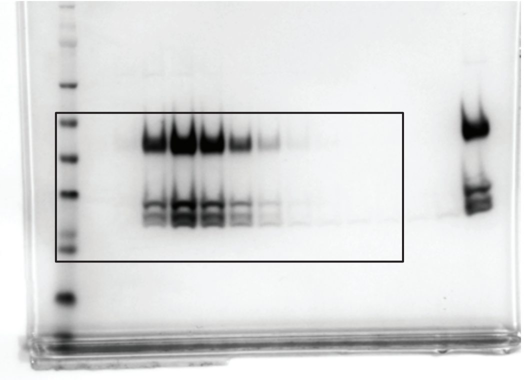

$MIS12C^{Nsl1\Delta C}$
Knl1

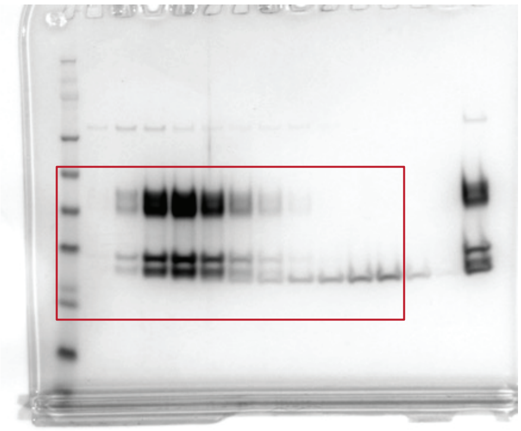

$MIS12C^{Nsl1\Delta C}$
$Knl1^{mut1}$

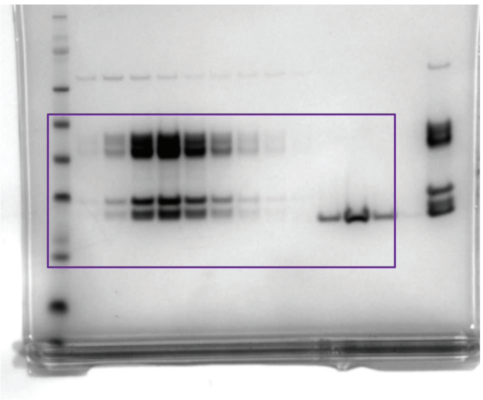

$MIS12C^{Nsl1\Delta C}$
$Knl1^{mut2}$

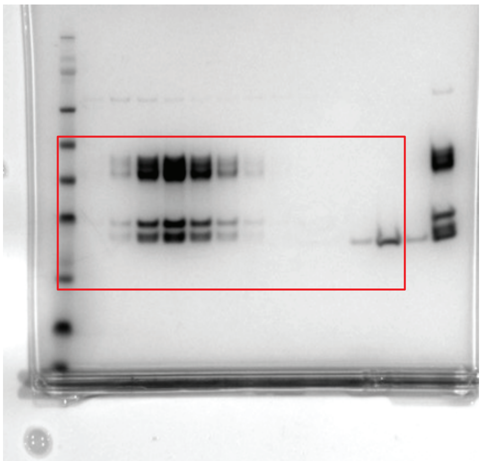

MIS12C

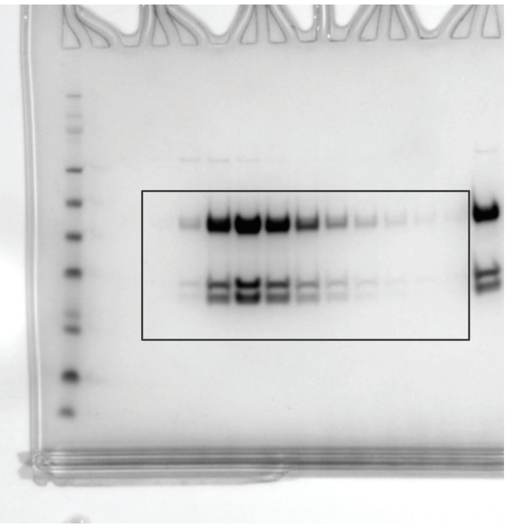

Knl1

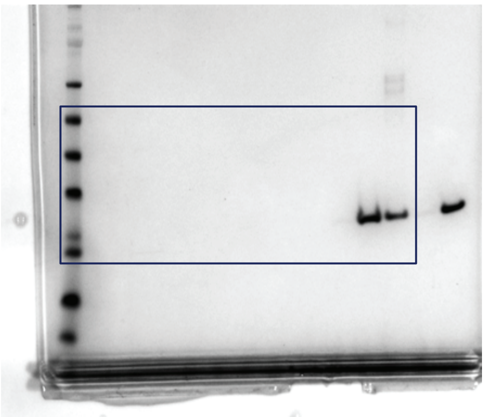

Supplement: Supplementary file 5 — Unprocessed SDS–PAGE gels, labeled for each extended data figure. [file 41594_2024_1249_MOESM5_ESM.pdf]
